# Fetomaternal Outcomes in Women Affected With Preterm Premature Rupture of Membranes: An Observational Study From a Tertiary Care Center in Eastern India

**DOI:** 10.7759/cureus.25533

**Published:** 2022-05-31

**Authors:** Neha Singh, Lipipuspa Pattnaik, Soumya R Panda, Pramila Jena, Jyochnamayi Panda

**Affiliations:** 1 Obstetrics and Gynecology, Kalinga Institute of Medical Sciences, Bhubaneswar, IND

**Keywords:** maternal morbidity, preterm premature rupture of membranes, feto-maternal outcome, chorioamnionitis, pprom

## Abstract

Introduction: Preterm premature rupture of membranes (PPROM) is the spontaneous rupture of the fetal membranes before the completion of 37 weeks of pregnancy. PPROM occurs in 3% of pregnancies.

Methodology: This prospective observational study was conducted between September 2019 and March 2021, involving 150 antenatal patients attending our outpatient department or labor room. All pregnant women with a singleton pregnancy between 28 and 37 weeks of gestational age with PPROM were included in our study.

Results: A total of 44% of women were admitted to the hospital within 6-11 hours of the onset of PPROM, while 34% of women were admitted within five hours and 15.33% were admitted within 12-23 hours of the onset of PPROM. The most common organisms isolated in high vaginal swabs were *Enterococcus faecalis* (18%), *Escherichia coli* (12%), *Staphylococcus aureus* (12.66%), *Staphylococcus haemolyticus* (6.66%), and *Candida albicans* (4.66%). Around 74.66% of women were delivered within 24 hours of the onset of PPROM, whereas only 2.6% of patients were delivered after 72 hours and the rest 34% were delivered between 25 and 72 hours. Of our study subjects, 10% were febrile, 4% were having urinary tract infections, 2.5% had postpartum hemorrhage, and 2% had chorioamnionitis. As far as neonatal morbidity and mortality are concerned, birth asphyxia and jaundice were seen in 12% of patients each, whereas septicemia was found in 4% of study subjects.

Conclusion: Owing to the association of higher maternal and perinatal morbidity and mortality, cases, especially in the early PPROM group, should be strictly monitored for clinical and laboratory signs of chorioamnionitis while opting for conservative management.

## Introduction

Preterm premature rupture of membranes (PPROM) is the spontaneous rupture of amniotic membranes prior to the onset of labor and before 37 completed weeks (36 weeks + six days) of gestational age [1]. PPROM has an incidence of 3% among all pregnancies and is associated with approximately one-third of all preterm births and 10% of perinatal death. PPROM complicates about 2-4% of singleton pregnancies and 7-20% of twin pregnancies. PPROM is an important cause of perinatal morbidity and mortality mainly due to prematurity and its sequelae. Maternal morbidity is increased because of chorioamnionitis, unfavorable cervix, dysfunctional labor, increase in cesarean section rates, postpartum hemorrhage (PPH), and endometritis. Perinatal infection, increased incidence of hyaline membrane disease, intraventricular hemorrhage, sepsis, cord prolapse, umbilical cord compression resulting from oligohydramnios, and fetal distress further compromise the outcome and there is increased fetal wastage [2]. The longer the time interval between the rupture of membranes and onset of labor, the greater the risk of ascending infections and chorioamnionitis. This risk may assume a grave prognosis in patients undergoing cesarean section.

With the association of significant maternal and neonatal adverse consequences, PPROM bears a significant dilemma in its management. Hence, careful consideration of various factors and individualization of cases is necessary for appropriate management [3]. The present study is undertaken to know the current trends of fetal and maternal outcomes associated with PPROM so that increased attention will be diverted to the important causative factors and fetomaternal outcomes can be improved.

## Materials and methods

This prospective observational study was conducted in the Department of Obstetrics and Gynecology, Kalinga Institute of Medical Sciences, Bhubaneswar, which is a tertiary care center catering to a major burden of antenatal care in eastern India. This study was carried out between September 2019 and March 2021 on antenatal patients attending our outpatient department (OPD) or labor room.

Inclusion criteria

All pregnant women with a singleton pregnancy between 28 and 37 weeks of gestational age with PPROM were included in the study.

Exclusion criteria

Complications of pregnancy other than PROM that affect fetal and neonatal outcomes, e.g. multiple pregnancies, fetal growth restriction, uterine anomalies, fetal anomalies, myoma uteri, hypertensive disorders and pregnancy-induced hypertension, gestational diabetes mellitus, and antepartum hemorrhage, were excluded from our study. Also, antenatal women affected with medical disorders of pregnancy, such as chronic renal failure and class II-IV cardiac diseases, were excluded from our study.

Methods

A detailed history and clinical examination were performed in all cases. The diagnosis of PPROM was established by history and sterile pelvic speculum examination showing amniotic fluid trickling from the cervix, pad test, routine hematological investigations, urine examination, high vaginal swab (HVS), cardiotocograph, and obstetric ultrasound examination. Ultrasonography was done to assess gestational age, growth parameters, presentation, exclusion of congenital anomalies, and amniotic fluid index.

Conservative management was done in all early PPROM (28 weeks to 33 weeks + six days) patients till the onset of spontaneous labor or till the maternal or fetal indication for delivery ensues such as chorioamnionitis, meconium-stained amniotic fluid, abruption, cord prolapse, fetal distress, and/or advanced labor on admission. All late PPROM (>34 weeks) patients were induced if not getting into spontaneous labor. Patients were hospitalized until delivery. Two doses of betamethasone (12 mg) at 12 hours apart or four doses of dexamethasone (6 mg) at 12-hour intervals were administered to the patients with <34 weeks of pregnancy to enhance fetal lung maturity. Prophylactic antibiotics were used in all cases for 10 days or up to delivery. Maternal monitoring to detect chorioamnionitis was done by monitoring pulse rate, temperature, abdominal tenderness, color and smell of liquor, and fetal tachycardia in cardiotocography. Mothers were monitored intrapartum for complications such as abruption, PPH, and retained placenta. Neonates with poor Apgar (appearance, pulse, grimace, activity, and respiration) scores, prematurity, or infection were admitted to the neonatal intensive care unit (NICU) for further management and their outcomes were studied. Follow-up visits were conducted for mothers for up to six weeks postpartum.

Statistical analysis

The data were tabulated and expressed as mean ± SD for continuous variables or frequency and percentage for categorical variables. Chi-square or Fisher's exact test was used to determine the association between two categorical variables. The Student’s t-test was performed to test the significance of the difference between the two groups. All statistical calculations were performed using SPSS version 21 (IBM Corp., Armonk, NY), and a p-value ≤ 0.05 was considered statistically significant.

## Results

A total of 150 patients were recruited for the study, out of which around 34% were booked cases. As shown in Table 1, almost half of the women (46.67%) were in the age group of 26-30 years while only 6% of women were in the age group of >35 years. The mean age was 26.64 years with a standard deviation of 4.33 years. Most of the women (68.7%) in our study were from lower socioeconomic status. While 47.33% of women in our study were in the gestational age bracket of 34-36 weeks + six days, 30% were between 28 and 31 weeks + six days, and the rest 22.67% were between 32 and 33 weeks + six days of gestational age (Table 1). A total of 44% of women were admitted to the hospital within 6-11 hours of the onset of PPROM, while 34% of women were admitted within five hours and 15.33% were admitted within 12-23 hours of the onset of PPROM (Table 1). Thus, the majority of women sought hospital admission at the earlier duration of onset of PPROM.

**Table 1:** Baseline characteristics PPROM: preterm premature rupture of membranes.

	Frequency	Percentage (%)
Age distribution		
20-25	36	24
26-30	70	46.67
31-35	35	23.33
>35	9	6
Socio-economic status		
Low	103	68.67
Middle	41	27.33
Upper middle	6	4
Gestational age		
28-31.6	45	30
32-33.6	34	22.67
34.36.6	71	47.33
Obstetrics status		
Primigravida	84	56
Multigravida	66	44
Time (in hours) between PPROM to the admission of study subjects		
0-5	51	34
6-11	66	44
12-23	23	15.33
24-47	7	4.67
47-73	3	2

As shown in Table 2, the most common organisms isolated in HVS were *Enterococcus faecalis* (18%), *Escherichia coli* (12%), *Staphylococcus aureus* (12.66%), *Staphylococcus haemolyticus* (6.66%), and *Candida albicans* (4.66%), in that order. In 16% of subjects, the culture and sensitivity did not reveal any growth.

**Table 2:** Organisms isolated on high vaginal swab culture MRSA: methicillin-resistant *Staphylococcus aureus.*

High vaginal swab	Frequency	Percentage
Acinetobacter baumannii	7	4.66
*Acinetobacter* spp.	1	0.66
Acinetobacter lwoffii	1	0.66
Candida albicans	7	4.66
Candida glabrata	1	0.66
Candida tropicalis	2	1.33
Citrobacter koseri	1	0.66
*Citrobacter *spp.	1	0.66
*Enterobacter cloacae* complex	1	0.66
*Enterobacter *species	1	0.66
Enterococcus faecalis	27	18
Enterococcus faecium	1	0.66
*Enterococcus *spp.	7	4.66
Escherichia coli	18	12
*Klebsiella pneumoniae* ssp. pneumoniae	6	4
Proteus mirabilis	1	0.66
Pseudomonas aeruginosa	5	3.33
Serratia marcescens	1	0.66
Staphylococcus aureus	19	12.66
*Staphylococcus aureus* (MRSA)	2	1.33
Staphylococcus epidermis	4	2.66
Staphylococcus haemolyticus	10	6.66
Staphylococcus saprophyticus	1	0.66
*Streptococcus agalactiae* (group B)	1	0.66
No growth	24	16
Total	150	100

As far as the latent period for delivery is concerned, 74.66% of women were delivered within 24 hours of the onset of PPROM, whereas only 2.6% of patients were delivered after 72 hours and the rest 34% were delivered between 25 and 72 hours (Table 3).

**Table 3:** Maternal morbidity

	Frequency	Percentage
Febrile morbidity	20	10.0
Postpartum hemorrhage	5	2.5
Urinary tract infection	8	4.0
Antepartum hemorrhage	1	0.5
Wound infection	2	1.0
Chorioamnionitis	3	2
Mode of delivery		
Vaginal delivery	101	67.33
Lower segment cesarean section	49	32.67
Latent period for delivery (in hours)		
0-24	112	74.66
25-72	34	22.67
>72	4	2.67

While the majority (67.33%) of the study subjects were delivered vaginally, 32.67% were delivered by lower segment cesarean section (LSCS). Out of all vaginal deliveries, 76% of patients were delivered spontaneously, whereas 61% of patients were induced. The most common indication was the failure to progress, accounting for 38.58%, and in another 34.54% of cases, the indication was fetal distress. C-reactive protein (CRP) was raised in 44% of cases, while raised total leukocyte count (TLC) was observed in 29.33% of cases. However, chorioamnionitis was observed only in 2% of study subjects. In our study subjects, around 10% of women were febrile, 4% were having urinary tract infections (UTIs), 2.5% had PPH, and 2% had chorioamnionitis (Table 3). As far as neonatal morbidity and mortality are concerned, birth asphyxia and jaundice were seen in 12% of patients each, whereas septicemia was found in 4% of study subjects (Table 4).

**Table 4:** Distribution of neonatal morbidity

Neonatal morbidity	Frequency	Percentage
Healthy	108	72
Birth asphyxia	18	12
Jaundice	18	12
Septicemia	6	4
Birth weight		
<1.5 kg	33	22
1.5-2.0 kg	19	12.67
2.0-2.5 kg	65	43.33
≥2.5 kg	33	22
Neonatal intensive care unit admission	50	33.33
Neonatal mortality	3	3.3

Of newborn babies, 43.33% weighed between 2 and 2.5 kg, whereas 22% of subjects had a birth weight of less than 1.5 kg. The NICU admission rate was found to be 33.33%, whereas there was neonatal mortality in 3.3% of babies (Table 4). The indicators of maternal morbidity, such as raised TLC, raised CRP, and operative interference, were increased in the gestational age group of 34-36 weeks and six days as compared to the gestational age group of 28-33 weeks and six days, which was statistically significant (p-value < 0.05). NICU admissions were significantly higher in early PPROM patients (Table 5).

**Table 5:** Distribution of selected morbidity factors in different gestational age GA: gestational age.

Morbidity factor	GA: 28-31 weeks + 6 days	GA: 32-33 weeks + 6 days	GA: 34-36 weeks + 6 days	P-value
Maternal				
Raised total leukocyte count	14	8	22	0.03455
Raised C-reactive protein	15	12	34	0.0009
Febrile illness and chorioamnionitis	8	5	6	0.6902
Operative interference	15	7	27	0.0020
Fetal				
Birth asphyxia	9	3	7	0.2289
Jaundice	2	8	7	0.1611
Septicemia	2	1	3	0.6065
Neonatal intensive care unit admission	28	16	6	0.0006

However, on multi-logistic regression analysis, we could not find any significant association of any particular parameter to unfavorable maternal or fetal outcomes (Tables 6, 7).

**Table 6:** Multi-logistic regression analysis of maternal outcomes and associated factors of pregnancy complicated by PPROM ANC: Antenatal check-up; PPROM: preterm premature rupture of membranes; TLC: total leukocyte count; CRP: C-reactive protein; VD: vaginal delivery; LSCS: Lower segment cesarean section.

Variables			Favorable	Unfavorable	P-value	OR (95% CI)
ANC follow-up	Yes, No	51, 99	39, 72	12, 27	0.457	0.73 (0.32-1.66)
Duration of PROM	<12 hours	117	89	28	0.505	0.73 (0.28-1.84)
	≥12 hours	33	11	12		
Latency	<24 hours	112	85	27	0.304	0.66 (0.30-1.44)
	≥24 hours	38	25	13		
Raised TLC	Yes	102	92	30	0.266	1.63 (0.68-3.90)
	No	48	38	10		
Raised CRP	Yes	66	43	23	0.081	2.08 (0.91-4.74)
	No	84	67	17		
Microbial growth on high vaginal swab	Yes	126	85	41	0.524	0.69 (0.22-2.11)
	No	24	14	10		
Mode of delivery	VD	101	79	22	0.386	0.69 (0.29-1.59)
	LSCS	49	30	19		

**Table 7:** Multi-logistic regression of fetal outcomes and associated factors of pregnancy complicated by PPROM ANC: antenatal check-up; PPROM: preterm premature rupture of membranes; CRP: C-reactive protein; VD: vaginal delivery; LSCS: lower segment cesarean section; Apgar: appearance, pulse, grimace, activity, and respiration.

Variables			Favorable	Unfavorable	P-value	OR (95% CI)
ANC follow-up	Yes	51	36	15	0.393	0.71 (0.32-1.54)
	No	99	63	36		
Duration of PPROM	<12 hours	117	79	38	0.118	0.48 (0.19-1.20)
	≥12 hours	33	20	13		
Latency	<24 hours	112	76	36	0.268	0.66 (0.32-1.37)
	≥24 hours	38	23	15		
Raised CRP	Yes	66	45	21	0.553	0.78 (0.35-1.73)
	No	84	54	30		
Apgar score at 5 minutes	<7	28	15	13	0.171	1.85 (0.76-4.50)
	≥7	122	84	38		
Birth weight	<2500 gm	117	79	38	0.182	0.56 (0.24-1.31)
	≥2500 gm	33	20	13		
Mode of delivery	VD	101	65	36	0.267	1.59 (0.69-3.63)
	LSCS	49	33	16		

## Discussion

This was a hospital-based observational, prospective study, conducted with 150 study subjects to evaluate the fetomaternal outcomes in PPROM cases. The majority of women (68.7%) in our study were from a lower socioeconomic status. The majority (47.33%) of study subjects belonged to 34-36 weeks and six days of gestational age similar to the study conducted by Addisu et al. [4], where 69.6% of women belonged to the gestational age of 34-36 weeks and rest 30.4% to 29-33 weeks. In the present study, 46.7% of women were admitted within 24-47 hours of PPROM, 44% within 6-11 hours of PPROM, and 34% within five hours.

Similar to the studies conducted by Patil and Patil [5] and Russell and Anderson [6], the majority of women (74.66%) in our study delivered within 24 hours of PPROM. Only 11% had a latent phase of more than three days, and 28.5% delivered within 25-72 hours in our study, which also correlated with the above-mentioned studies. In a study by Sultana and Karmokar [7], the mean time interval of onset of membrane rupture and delivery was 27.60 hours with a standard deviation of 21.12 hours. In another study [8], the mean time of delivery after admission to the hospital was 37.13 ± 17.43 hours in patients with PPROM.

In the present study, the most common organism found in HVS culture was *Enterococcus faecalis* (18%), followed by *Escherichia coli* (12%), *Staphylococcus aureus* (12.66%), *Staphylococcus haemolyticus* (6.66%), and *Candida albicans* (4.66%). Although a similar group of organisms was also found in a study by Pandey et al. [9], the most common organism isolated was *E. coli*, followed by *Staphylococci*, *Streptococci*, and atypical coliforms.

In our study, the majority (67.33%) of the study subjects were delivered vaginally. Among them, 55.66% of the study subjects delivered spontaneously while 45.66% were induced. Other studies also reported similar incidences of vaginal deliveries [7,10-12].

The rate of cesarean deliveries in our study was 32.67%. The most common indication was a failure to progress (38.58%), followed by fetal distress (34.54%). This is in contrast to other studies [9,13-15], where the most common indications for LSCS were fetal distress, breech presentation, and chorioamnionitis, respectively. In our study population, 44% of women were found to be CRP positive. However, the incidence of raised CRP was found to be 24% by Ashraf et al. [11], and 11% by Mohan et al. [14]. While only 2% of our study subjects had chorioamnionitis, there are reports of a comparatively higher incidence of chorioamnionitis by Rajan and Menon (13%) [13] and Ashraf et al. (11%) [11]. This could be due to better antenatal care and timely delivery in our tertiary care hospital.

The incidence of various maternal morbidity factors in our study, such as febrile illness (10%), UTI (4%), PPH (2.5%), chorioamnionitis (1.5%), and wound infections (1%), was comparatively lower than other reported studies [4,13,15]. The incidence of low birth weight and very low birth weight babies in the present study was 43.33% and 22%, respectively. In our study, 33.33% of neonates were admitted to NICU and the NICU admissions were noted to be significantly higher in the early PPROM group. Birth asphyxia was seen in 12% of patients, jaundice in 12%, and septicemia in 4% of the subjects, and the neonatal death rate was 3.33%. Patil and Patil [5] showed a similar incidence of NICU admission (36%). The studies by Poovathi et al. [10], Petit et al. [16], and Rajan and Menon [13] also found similar incidences of neonatal morbidity and mortality. Mohan et al. [14] observed severe respiratory distress syndrome in 43% of neonates and sepsis in 24% of cases. Analyzing various morbidity outcomes in PPROM in different gestational age groups, we observed that TLC and CRP were significantly raised in the 34-36 weeks and six days age group followed by the 28-31 weeks and six days age group. Cesarean section rates were also significantly higher in the same group. Febrile illness and chorioamnionitis were found most commonly at the gestational age of 28-31 weeks and six days, though the finding is not statistically significant because of the smaller sample size and a significant increase in NICU admission in early PPROM patients (28-31 weeks) (p-value < 0.05).

Thus, the earlier the gestational age at the time of PPROM, the longer the latency with more complications. In planning the management, several issues need to be considered. Prematurity is the principal risk to the fetus while infectious morbidity is the primary maternal risk. Chorioamnionitis with PPROM is responsible for significant maternal and neonatal morbidity including early-onset neonatal sepsis, bronchopulmonary dysplasia, intraventricular hemorrhage, and periventricular white matter injury [3].

On multi-regression analysis, there was no significant association with any particular parameter to maternal or fetal outcome. Hence, we suggest multiple factors should be taken into account while managing such cases of PPROM. This particular finding also directs future research studies to consider multiple combinations of factors and construct various mathematical models, which could help predict the maternal and fetal outcomes of PPROM cases.

## Conclusions

To conclude, there is an increased incidence of maternal and perinatal morbidity and mortality in patients with PPROM. Owing to the association of higher infective morbidity, cases, especially in the early PPROM group, should be strictly monitored for clinical and laboratory signs of chorioamnionitis while opting for conservative management. One needs to balance the risk of the development of chorioamnionitis against the risk associated with the delivery of a premature baby while taking crucial decisions regarding the timing of termination of pregnancy. According to our study, no particular factor could determine the fetomaternal outcomes and hence a combination of various clinical and laboratory parameters should be considered while making a decision of delivery. We suggest future research to design a special model taking a combination of various factors in predicting the outcome of pregnancies with PPROM.
